# Monitoring of dynamic ATP level changes by oligomycin-modulated ATP synthase inhibition in SW480 cancer cells using fluorescent “On-Off” switching DNA aptamer

**DOI:** 10.1007/s00216-019-02061-0

**Published:** 2019-08-12

**Authors:** Katarzyna Ratajczak, Agnieszka Lukasiak, Hubert Grel, Beata Dworakowska, Slawomir Jakiela, Magdalena Stobiecka

**Affiliations:** Department of Biophysics, Warsaw University of Life Sciences (SGGW), 159 Nowoursynowska Street, 02776 Warsaw, Poland

**Keywords:** Adenosine triphosphate, ATP aptamer, Oligomycin, ATP synthase inhibition, Intramolecular fluorescence resonance energy transfer (iFRET), Microscopic images

## Abstract

Adenosine triphosphate (ATP) is the main energy source in cells and an important biomolecule participating in cellular reactions in living organisms. Since the ATP level changes dynamically reflecting the development of a debilitating disease or carcinogenesis, we have focused in this work on monitoring of the oligomycin (OMC)-modulated ATP synthase inhibition using a fluorescent-switching DNA aptamer designed for the detection of ATP (Apt(ATP)), as the model for studies of dynamic ATP level variation. The behavior of the ATP aptamer has been characterized using fluorescence spectroscopy. The Intramolecular fluorescence resonance energy transfer (iFRET) operates in the proposed aptamer from the FAM dye moiety to guanines of the aptamer G-quadruplex when the target ATP is present and binds to the aptamer changing its conformation. The iFRET process enables the detection of ATP down to the limit of detection, LOD = 17 μM, without resorting to any extra chemi-amplification schemes. The selectivity coefficients for relevant interferent triphosphates (UTP, GTP, and CTP) are low for the same concentration as that of ATP. We have demonstrated an efficient transfection of intact cells and OMC-treated SW480 colon cancer cells with Apt(ATP), using microscopic imaging, iFRET measurements, and cell viability testing with MTT method. The applicability of the switching DNA aptamer for the analysis of real samples, obtained by lysis of SW480 cells, was also tested. The proposed Apt(ATP) may be considered as a viable candidate for utilization in measurements of dynamic ATP level modulation in cells in different stages of cancer development and testing of new drugs in pharmacological studies.

**Electronic supplementary material:**

The online version of this article (10.1007/s00216-019-02061-0) contains supplementary material, which is available to authorized users.

**Figure:**
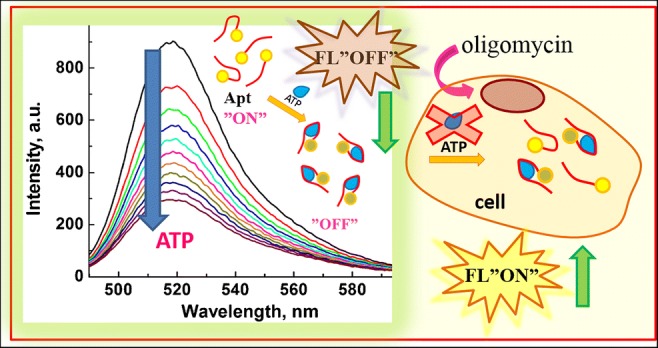
Graphical abstract

## Introduction

Adenosine triphosphate (ATP) is the key molecular energy supplier for cellular reactions [1, 2]. The deviations in ATP level have been attributed to serious diseases, including cardiovascular diseases [3], Huntington’s disease [4], hypoxia [5, 6], and inflammatory diseases [7]. The depletion of intracellular ATP level has been found to promote the necrosis pathway in cells over the apoptosis process [8–10]. On the other hand, the increased extracellular ATP level is characteristic for the tumor sites and its microenvironment [11–14]. Recently, it was also shown that the ATP acts as a hydrotrope to help solubilize hydrophobic proteins [15]. Therefore, monitoring of ATP content in living cells has become an important tool for characterization of cell homeostasis and assessment of ATP production in different stages of the diseases [16, 17]. Consequently, sensitive and inexpensive analytical methods for probing of intracellular ATP levels are highly desired.

Among many methods of ATP determination reviewed recently [14, 18–20], the methods based on ATP aptamer molecular beacons are the most convenient, sensitive, and biocompatible. The unique features of aptamers are due to the inherent nucleic acid biorecognition ability and sensitive fluorescence resonance energy transfer (FRET) detection system [21]. Owing to these features, the aptamer molecular beacons find diverse applications in many fields. Recently, the fluorescein-labeled aptamer was utilized for the detection of one of the most toxic mycotoxins aflatoxin B_1_ (AFB_1_) [22]. AFB_1_ was also detected by Ye and coworkers [23] using fluorescence polarization (FP) assay with graphene oxide for signal enhancement. Su et al. [24] have utilized the FAM–aptamer–G-quadruplex construct for the detection of organic compound melamine widely applied in manufacturing of plastics. Hence, the sensing platforms for ATP detection based on aptamers have been selected for this investigation.

The DNA and RNA sequences for specific ATP binding were first introduced by the Szostak’s group [25, 26] in the 1990s. Since that time, numerous modifications of ATP aptamers with fluorescent or electrochemical tags were proposed for the design of biosensors [27], as well as the electroluminescence [28] and chemiluminescence assays [29–31] for the detection of ATP. Exemplary aptasensors for sensitive detection of ATP down to pM range are presented in Table 1. With the variety of sensing systems available for ATP determination, the selection of the sensing platform depends largely on the conditions of the analysis. The methods offering ultra-high sensitivities may be useful when high sample dilution is required due to the biological matrix effects. However, for real-time sensing in cells, less sensitive but robust systems possibly based on a single probe molecule would be required. The latter approach is especially desired since the intracellular ATP content is often very high and can reach up to 150 mM [47].

**Table 1:** Sensitive aptasensors for ATP detection

No.	Oligonucleotide sequence for ATP targeting	Linearity/LOD	Buffer	Method	Ref.
1	**ADNA**: 5′-TCA CAG ATG AGT TT-SH-3′ **RDNA**: 5′-HS-CCC AGG TTC TCT-3′ **TRDNA**: 5′-ACT CAT CTG TGA AGA GAA CCT GGG GGA GTA TTG CGG AGG AAG GT-3′	Linear range 1 nM to 10 μM LOD = 0.2 nM	10 mM Tris-HCl, pH 7.4 + 50 μM [Ru(NH_3_)_6_]^3+^	Electrochemical	[32]
2	**ABA** (5′-ACC TGG GGG AGT ATT GCG GAG GAA GGT-3′) **FITC-labeled ABA** (F-ABA, 5′-FITC-ACC TGG GGG AGT ATT GCG GAG GAA GGT-3′)	LOD = 50 nM	20 mM phosphate buffer solution (pH 7.4)	Colorimetric	[33]
3	5′-CTCTCACCTGGGG GAGTATTGCGGAGGAAGGT-FAM-3′	Linear range 10–800 nM LOD = 4.5 nM	20 mM Tris-HCl (146 mM NaCl, 5.0 mM KCl, 1.0 mM MgCl_2_, pH 7.4)	Fluorimetric	[34]
4	**5′-thiol-modified aptamer**: 5′-TTT TTT ACC TGG GGG AGT ATT GCG GAG GAA GGT-3′	Linear range 0.2–10.0 μM LOD = 0.1 μM	16.0 mM Tris-HCl (15.0 mM KCl, 300.0 mM NaCl, pH 7.4)	Colorimetric	[35]
5	5′-HS-(CH_2_)_6_-ACCTGGGGGAGTATTGCGGAGGAAGGT 5′-biotin-ACCTTCCTCCG CAATACTCCCCCAGGT	Linear range 0.018–90.72 μM LOD = 6 nM	100 mM Na_2_HPO_4_ + NaH_2_PO_4_, 5 mM MgCl_2_, pH 7.4, and abbr. PBS+	Electrochemical	[36]
6	**ATP binding aptamer**: 5′-ACCTGGGGGAGT ATTGCGGAGGAA GGT-3′	Linear range 0.1–60 μM LOD = 33 nM	20 mM Tris-HCl, 5 mM MgCl_2_, and 300 mM NaCl, pH = 8.3	Fluorimetric	[37]
7	**ABA**: 5′-ACCTGGGGGA GTATTGCGGAGGAAGGT-3′ **cDNA**: 5′-NH_2_-(CH_2_)_6_-ACCTTCC TCCGCAATACTCCCCCAGGT-3′	Linear range 100 nM–5 mM LOD = 14.2 nM	20 mM PBS, (100 mM NaCl, 10 mM KCl, 1 mM MgCl_2_, pH 7.4)	Fluorimetric	[38]
8	**Oligonucleotide** 5′-TAACCCCTA ACCCCT-3′	Linear range 0.1–10 μM LOD = 33 nM	10 mM PBS pH 3.0	Fluorimetric	[39]
9	**ATP-binding aptamer**: 5′-AAC CTG GGG GAG TAT TGC GGA GGA AGGT-3′ **Complementary strand**: 5′-ACC TTC CTC CGC AAT ACT CCC CCA GGTT-3′	Linear range 0.5–50 μM LOD = 140 nM	20 mM Tris-HCl (2 mM MgCl_2_, pH 8.3)	Fluorimetric	[40]
10	**DNA**_**1**_: 3′-SH-(CH_2_)_6_-TGG AAG GAG GCG TTA TGA GGG GGT CCA-5′ **DNA**_**2**_: 3′-AA CGC CTC CTT CCA-FAM-5′	Linear range 0.1–10 mM LOD = 15.2 nM	10 mM PBS (0.1 M NaCl, pH = 7.0)	Fluorimetric	[41]
11	**MB-like DNA**: 5′-HS-(CH2)6-CCT CTC CGT GTC TTG TAC TTC CCG TCA GAG A GGbiotin-3′ **Oligo A**: 5′-p-TAC AAG ACA C-3′ **Oligo B**: 5′-GAC GGG AAG-3′	Linear range 0.1–1000 nM LOD = 0.05 nM	20 mM Tris-HCl (5 mM MgCl_2_)	Electrochemical	[42]
12	**ABA**: 5′ATGTCACCTGGGGGAGTATTGCGGAGGAAGGTCTGTA 3′ **ROS**: 5′ATGTCACCTGGGGGAAGGTCTGTA 3′ **PCS1**: 5′HS-(CH_2_)_6_-TACAGACCTTCC 3′ **PCS2**: 5′ CCCAGGTGACAT-(CH_2_)_6_-SH 3′	Linear range 0.1–100 nM LOD = 0.1 nM	25 mM Tris-HCl (100 mM NaCl, pH 8.2)	Electrochemical	[43]
13	**ABA**: 5′-HS-(CH_2_)_6_-ACC TGG GGG AGT ATT GCG GAG GAA GGT-3′ **BCS**: 5′-biotin-ACC TTC CTC CGC AAT ACT CCC CCA GGT-3′	Linear range 0.01–100 nM LOD = 0.01 nM	10 mM PBS (0.1 M KCl, 2 mM Fe(CN)_6_^3−/4−^, pH 7.4)	Electrochemical	[44]
14	**Aptamer 1 (Apt 1)**: TTTTTTTTTTTTTTTTTTACCTGGGGGAGTAT **Aptamer 2 (Apt 2)**: TGCGGAGGAAGGTTTTTTTTTTTTTTTTTTT	Linear range 100 nM–100 μM LOD = 10.29 nM	10 mM Tris-HCl (10 mM MgCl_2_, 50 mM NaCl, pH 7.9)	Fluorimetric	[45]
15	**TBA**: 5′-GTGGTAGGGC AGGTTGGGGTGACT-3′ **cTBA**: 5′-GTGTGTAGTCA CCCCAACCTGCCC-3′ **ABA**: 5’-ACCTGGGGGAGTA TTGCGGAGGAAGGT-3′ **cABA**: 5′-CTTCCTCCGCAAT ACTCCCCCAGGT-3′	LOD = 23.4 nM	50 mM Tris-HCl (100 mM NaCl, 10 mM MgCl_2_, pH 7.5)	Fluorimetric	[46]

Since a model system mimicking biochemical processes leading to ATP generation is required for monitoring of dynamic changes in ATP production assessment, we have developed a system based on SW480 colon cancer cells with modulation of ATP synthase inhibition by exogenous oligomycin (OMC). In cells, ATP is mainly produced by ATP synthase [48, 49]. This enzyme is also involved in the major pathway of proton flow into the mitochondrial matrix. To our knowledge, there are no publications on the effect of oligomycin (OMC) on ATP contents in living cells studied using fluorescent Apt(ATP) biosensing techniques. In recent studies, the ROS generation and uncoupling of the Warburg effect in SW480 cells have been achieved by treatment with a natural flavonoid morin [50] and a decrease in GSH and ATP levels have been found using HPLC analysis applied to cell lysate. A selective detection of ATP in mitochondria and lysosomes has been reported by Swamy et al. [51] using fluorescent probes of rhodamine derivatives with thiourea.

In the present work, our investigations have focused on exploring the utilization of fluorescent “On-Off” switching DNA aptamer for monitoring of ATP in untreated and OMC-treated SW480 cancer cells. In our aptaprobe designs, we have employed a fluorescein (FAM)-labeled DNA aptamer (Apt(ATP)) without any further sensitivity amplification. The oligomycin-modulated ATP synthase inhibition was investigated using intramolecular fluorescence resonance energy transfer (iFRET), microscopic imaging, oxygen consumption measurements, and MTT viability test. In the presence of ATP, the aptamer changes conformation and binds with ATP forming an aptamer-ATP complex showing strong FAM fluorescence quenching due to the proximity to guanine nucleobase in the new aptamer conformation. We have analyzed the specificity and selectivity of the Apt(ATP) against interferents of similar structure, such as GTP, UTP, and CTP. The utility of the developed aptaprobes for detection of ATP in SW480 cell lysate samples was also evaluated. The proposed method has a potential for utilization in measurements of cellular ATP production in different stages of disease such as cancer development.

## Materials and methods

### Chemicals

Fluorescein (FAM)-labeled DNA aptamer for the recognition of ATP with the following sequence 5′-TCTCTCACCTGGGGGAGTATTGCGGAGGAAGGT-FAM-3′ has been synthesized by the Laboratory of DNA Sequencing and Oligonucleotides Synthesis, Institute of Biochemistry and Biophysics of the Polish Academy of Sciences (IBB PAS, Warsaw, Poland). The purity of this oligonucleotide was tested with HPLC. Oligomycin A, adenosine 5′-triphosphate disodium salt hydrate grade I (ATP), guanosine triphosphate (GTP), cytidine 5′-triphosphate disodium salt (CTP), uridine 5′-triphosphate trisodium salt dehydrate (UTP), trizma hydrochloride (Tris-HCl), hydrogen peroxide solution (H_2_O_2_), magnesium chloride (MgCl_2_), carbonyl cyanide 4-(trifluoromethoxy)phenylhydrazone (FCCP) and sodium chloride (NaCl), 0.25% Trypsin-EDTA solution were obtained from Sigma-Aldrich Chemical Company (St. Louis, MO, USA).

Fetal bovine serum (FBS) was obtained from Gibco (Rockville, MD, USA). Penicillin/streptomycin and Dulbecco’s modified Eagle’s Medium (DMEM) were obtained from the cell culture company PAA (Immuniq, Warsaw, Poland). All chemicals were of analytical grade purity. Aqueous solutions were prepared with freshly deionized water with 18.2 MΩ cm resistivity (Millipore, Poland). All concentrations of added reagents cited in this paper are final concentrations obtained after mixing, unless otherwise noted.

### Apparatus

The fluorescence spectra were recorded using Spectrometer model LS55 (PerkinElmer, Waltham, MA, USA), with 20 kW pulsed Xenon light source and a photomultiplier tube detector. The excitation and emission slit widths were set to 5.0 nm and scan speed to 500 nm/min. The measurements were performed in 20 mM Tris-HCl buffer + 100 mM NaCl + 5 mM MgCl_2_ solutions with pH 7.4. The excitation and emission wavelengths were set to *λ*_ex_ = 480 nm and *λ*_em_ = 516 nm, respectively.

The images of fluorescence emission of the aptamer probe from transfected cells were acquired with a Nikon Eclipse TE300 inverted light microscope with a B-2A fluorescence filter with a 30–50 nm bandwidth excitation filter, long-pass dichromatic mirror, and long-pass barrier filter. The images were recorded digitally using a Canon Power Shot A640 scope-mounted camera. All images were made with the same exposure. The images were then imported into Adobe Photoshop Elements 2019 for enhancement of lighting by adjusting the Levels function for green channel to clear the background below level 17 and saturating the high light above the level 141.

The cell oxygen consumption after addition of oligomycin to SW480 cell culture was measured at 37 °C using a Clark-type electrode and an Oroboros-2k high-resolution respirometer (Oroboros, Innsbruck, Austria).

The calculations of polymorphic structures of Apt(ATP)s, their folding energies, and melting temperatures were performed using the University of Albany web server DINAMelt providing the program UNAFold ver. 3.9 with a Quikfold application (RNA Institute, University of Albany, Albany, NY, USA).

### Cell culture

The experiments with oligomycin-modulated ATP synthase inhibition were performed using SW480 cell line purchased from ATCC (LGC Standards Sp. z.o.o., Lomianki, Poland). The cells were cultured in DMEM (Dulbecco’s modified Eagle’s Medium) with added 10% FBS (fetal bovine serum, Gibco, Rockville, MD, USA) and were maintained in a humidified air atmosphere containing 5% CO_2_ at 37 °C (Shel Lab Model 2123-TC CO_2_ Incubator Cornelius, OR, USA). Every 2–3 days, the cells were further subcultured. The cells remaining after the experiments were handled according to safety protocols.

### Cells transfection with Apt(ATP) @LIP

Transfection experiments were conducted on SW480 cells using commercial transfer agents Lipofectamine® LTX and PLUS™ Reagent (Life Technologies, USA) which formed liposomes able to cross a cell membrane. In the presence of Apt(ATP), these agents formed liposomes loaded with ATP aptamer (Apt(ATP)@LIP). To deliver ATP aptamers into the SW 480 cells, 521 μL of transfection solution containing aptamer, Lipofectamine, Plus Reagent solutions, and DMEM were added to the cells (1.6 × 10^5^ cells/mL), so that the final concentration of aptamer was 49 nM. Different concentrations of oligomycin (final concentration 1 or 5 μM) were added to the cells 20 h before the transfection of cells with Apt(ATP)@LIP. After adding the transfection solution, cells were incubated for 2 h at 37 °C, followed by washing and resuspension in the DMEM (3.5 mL) and recording of cell images. Next, a trypsin solution was added (0.5 mL, 0.25% solution) to detach cells and fluorescence measurements were performed. For the data collection and analysis, a PerkinElmer FL WinLab™ software was used.

### Cell viability

MTT test was performed to determine the cell viability following transfection of SW480 cells with ATP synthase inhibitor, oligomycin (OMC). Typically, cells (1.6 × 10^5^ cells/mL) were incubated in 2 mL culture medium containing OMC with different concentrations (0, 0.3, 1, and 5 μM) for 20 h. After the incubation, an aliquot of 100 μL of 5 mg/mL of yellow MTT reagent was added to the SW480 cells in a CO_2_ incubator. The tetrazolium salt MTT is reduced in the mitochondria of metabolically active cells to a formazan product enabling to evaluate the cells’ viability. After 3 h of exposure, the cells were detached from the culture dish by trypsin and centrifuged at 153 rcf to pellet the cells. Then, 1500 μL of a lysis buffer containing dimethyl sulfoxide and ethanol (DMSO: EtOH = 1:1) was added to 500 μL of cell suspension to dissolve the insoluble purple formazan product. After 15 min of incubation at 37 °C, the samples were diluted with Tris-HCl buffer and absorbance measurements at 570 nm were performed.

### Measurements of oxygen consumption

The measurements of oxygen consumption after the addition of oligomycin were performed using the procedure reported earlier [52]. Briefly, after trypsinization, SW480 cells were harvested by centrifugation at 200*g* for 5 min, resuspended in serum-free culture medium at a concentration of 1 × 10^6^ cells/mL, and placed in testing chambers.

The solutions of oligomycin tested (0.3 μM and 1 μM) were added to the chambers after the respiratory flux had been stabilized. Next, after the mitochondrial oxygen consumption depletion, FCCP (1 μM) was added as a positive control to uncouple oxidative phosphorylation. Results are presented as means ± S.E.M. Paired *t* tests were performed to evaluate the differences before and after addition of compounds. A value of *P* < 0.05 was accepted as being significant. The number of experiments (*n* = 3) means three separate experiments.

### Cell lysis

To obtain the cells lysate, ca. 1.6 × 10^5^ cells/mL were washed with PBS buffer and 1 mL of trypsin was added for 15 min at 37 °C for detaching of cells from the culture dish. Then, the centrifugation at 1600 rpm for 10 min was performed and the supernatant was discarded. Then, 1000 μL of a solubilization solution containing 20% (w/v) SDS (200 mg/mL), 50% (v/v) DMF (36.55 mg/mL), 2% (v/v) acetic acid, and 25 mM HCl was added. Next, 10 μL of cell lysate with final dilution 1:300 was added to measurement buffer (20 mM Tris-HCl, 100 mL NaCl, 5 mM MgCl_2_) with Apt(ATP). Then different concentrations of ATP (33.3, 66.7, 100, and 133.3 μM ATP, final concentration) were added and fluorescence measurements at excitation wavelength *λ*_ex_ = 480 nm were performed.

## Results and discussion

### Detection of ATP using fluorescent ATP aptamer

**Fig. 1:**
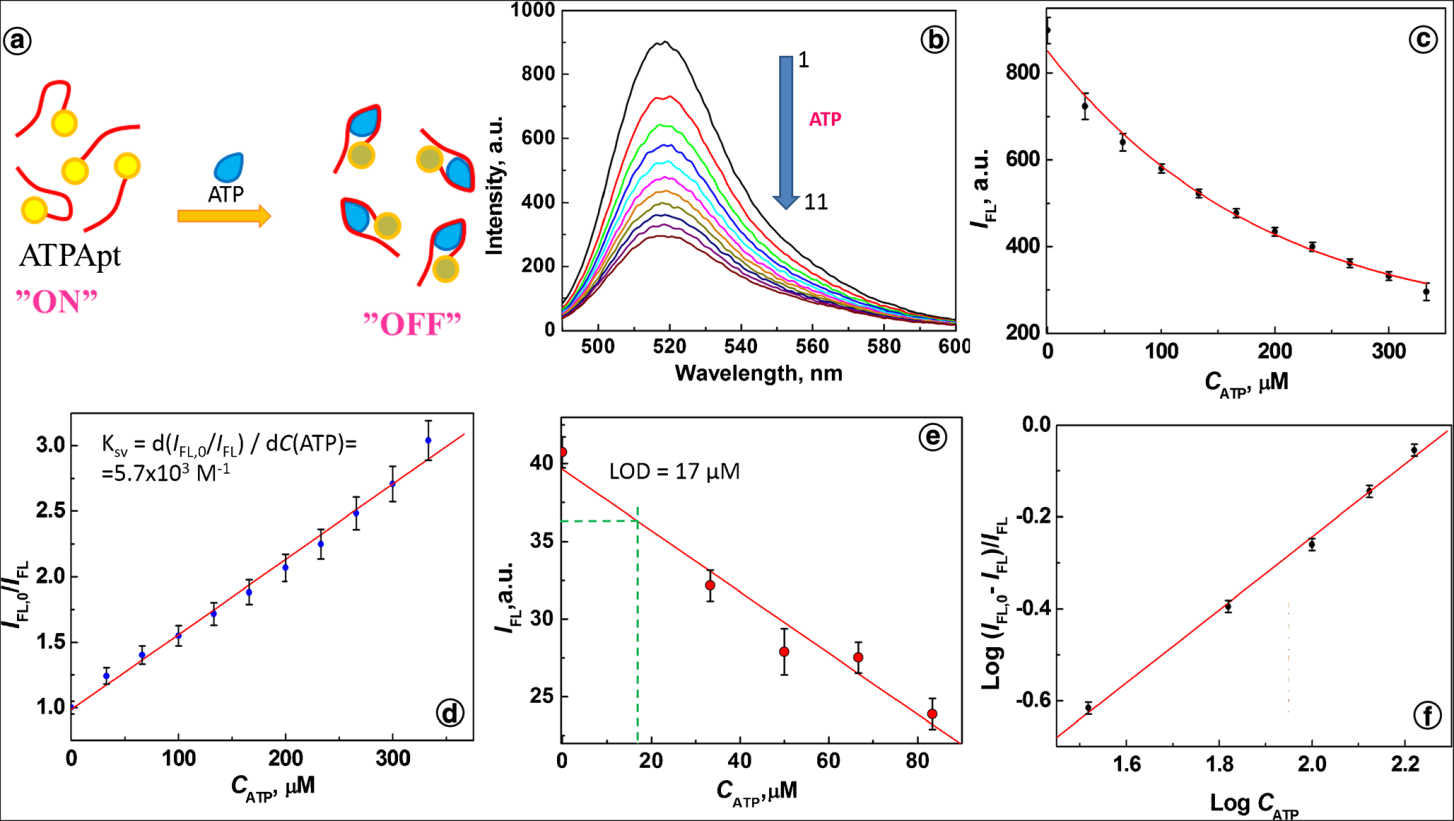
**a** Principle of ATP aptamer operation. **b** Fluorescence spectra for ATP aptamer after addition of ATP at different concentrations, *C*_ATP_ (mM): (1) 0, (2) 0.033, (3) 0.066, (4) 0.1, (5) 0.13, (6) 0.16, (7) 0.2, (8) 0.23, (9) 0.26, (10) 0.3, (11) 0.33. **c** Dependence of *I*_FL_ vs. *C*_ATP_. **d** Stern-Volmer plot. **e** Dependence of *I*_FL_ vs. *C*_ATP_ for *C*_Apt_ = 16.7 nM. **f** Dependence of log (*I*_FL,0_ - *I*_FL_)/*I*_FL_ vs. log *C*_ATP_; conditions: *C*_Apt_ = 66.7 nM; 20 mM Tris-HCl with 100 mM NaCl and 5 mM MgCl_2_, pH 7.4; *λ*_ex_ = 480 nm, *λ*_em_ = 516 nm

The mechanism of operation of ATP aptamer (Apt(ATP)) is depicted in Fig. 1a. In the presence of ATP, the aptamer binds with ATP forming an aptamer-ATP complex, and exhibits strong FAM fluorescence quenching by intramolecular fluorescence resonance energy transfer (iFRET). In a Tris-HCl buffer solution of pH 7.4, corresponding to the intracellular pH of SW480 cells, the Apt(ATP) shows a fluorescence emission with maximum at *λ*_em_ = 516 nm for excitation at *λ*_ex_ = 480 nm, as illustrated in Fig. 1 b, curve 1. Upon the addition of adenosine triphosphate solution (Fig. 1b, curves 2 to 11), the fluorescence signal of Apt(ATP) is strongly quenched due to the expected interactions of Apt(ATP) with ATP. The conformation of Apt(ATP) is changed and iFRET from the FAM dye moiety to guanines of the aptamer G-quadruplex occurs. The quenching efficiency approaches 67%, as the ATP emission intensity decreases steadily with increasing *C*_ATP_ from *I*_FL,0_ = 898.2 a.u. to *I*_FL,11_ = 295.5 a.u. (Fig. 1c). The quenching function *I*_FL,0_/*I*_FL_ is linear for the concentration range from 0 to 333 μM ATP as indicated in the Stern-Volmer plot in Fig. 1 d. The slope of the fitting line in this figure represents the quenching constant *K*_SV_, according to the Stern-Volmer equation:

1 $$ {I}_{\mathrm{FL},0}/{I}_{\mathrm{FL}}=1+{K}_{\mathrm{SV}}\ {C}_{\mathrm{Q}} $$

where the quencher concentration *C*_Q_ is given by the concentration of ATP: *C*_Q_ = *C*_ATP_. The value of *K*_SV_ determined from the experimental data is *K*_SV_ = (5.7 ± 0.2) × 10^3^ M^−1^.

The limit of detection LOD for adenosine triphosphate, determined by iFRET from the dependence of fluorescence quenching of Apt(ATP) on the ATP concentrations by a 3σ method, is LOD = 24 μM. A higher sensitivity of Apt(ATP) was achieved by lowering the aptamer concentration to 16.7 nM. Under this condition, LOD = 17 μM was obtained, as shown in Fig. 1 e. These results indicate a good sensitivity of Apt(ATP) toward ATP and this sensing method is comparable with recently published results for single-component ATP probes [53–55].

The size of the binding site (*n*) between Apt(ATP) and ATP was determined from the slope of the logarithmic plot (Fig. 1f):

2 $$ \log\ \left[\left({I}_{\mathrm{FL},0}-{I}_{\mathrm{FL}}\right)/{I}_{\mathrm{FL}}\right]=\log\ {K}_{\mathrm{b}}+n\ \log {C}_{\mathrm{Q}} $$

The plot provides the value of the binding site size, *n* = 0.8, indicating that the ratio of aptamers to ATP molecules in a complex is approximately 1:1. This is consistent with the data obtained by other researchers [26, 29, 56]. Upon the interaction with ATP, aptamer changes conformation and assumes a new tertiary structure due to the intramolecular and intermolecular interactions such as aromatic stacking and hydrogen bonding between Apt and ATP molecules [29, 57]. According to the works of Szostak and coworkers [26], the ATP molecule is bound in a binding pocket of the aptamer above the G-quadruplex and between two adenines belonging to the two free oligonucleotide chains. The conformational changes after the interactions with ATP were also observed for DNA three-way junction system [56].

A response of an Apt(ATP) probe to the higher concentrations of ATP, in the millimolar range, is presented in Fig. S1 in the Electronic Supplementary Material (ESM). The measurements under these conditions are characterized with a lower probe sensitivity, necessary to cover the larger dynamic range, including the elevated ATP levels in some cells under extreme conditions.

### Initial aptamer conformations in the absence of target ATP

**Fig. 2:**
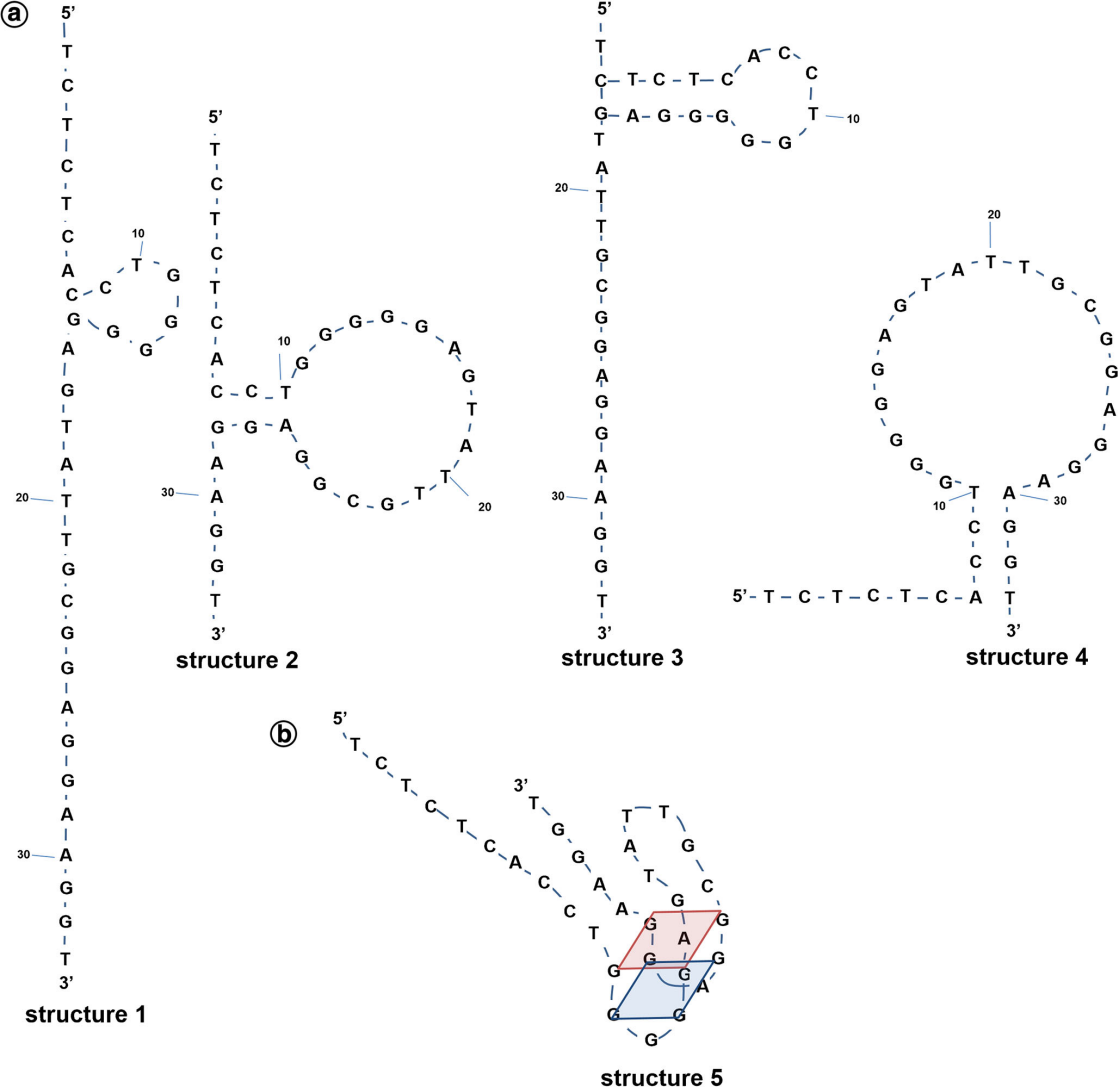
**a** Four secondary structures of Apt(ATP) obtained in 100 mM NaCl with 5 mM MgCl_2_ solution at 25 °C using UNAFold program. **b** Tertiary structure of Apt(ATP)

The potential secondary structures of the oligonucleotide aptamer for ATP (Apt(ATP)), in the absence of the ATP ligand, have been studied using UNAFold software [58, 59]. The obtained results are presented in Fig. 2a. For Apt(ATP) sequence used in the experiments and without FAM fluorescence dye, for 100 nM Apt(ATP) in 100 mM NaCl with 5 mM MgCl_2_ solution at 25 °C, four structures of Apt(ATP) were found. All structures had a negative Gibbs free energy of formation. The most thermodynamically stable was structure 1 with ΔG° = − 1.7 kcal/mol. The calculated thermodynamic data for the formation of conformational structures of Apt(ATP) and the respective equilibrium constants obtained from the dependence Δ*G*^0^ = -*RT*ln*K* are presented in Table 1. The tertiary structure of ATP aptamer designed according to Szostak et al. [26] and used in experiments is depicted in Fig. 2 b. The structures 2 and 4 may switch directly to the tertiary structure upon interaction with target ATP because their loops are composed of G-rich sequences which can fold into the characteristic G-quadruplex structure.

It follows from the analysis of thermodynamic data in Table 2 that Apt(ATP) structure 1 is predominant in a salt solution and it is also the most resistant to the formation of the G-quadruplex necessary to bind ATP ligands. The likely mechanism of ATP binding by the aptamer is thus via transition of Apt 1 to Apt 2, binding ATP, and completing the conformation change to structure 5.

**Table 2:** Thermodynamic data for the formation of conformational polymorphic structures of Apt(ATP) calculated for 100 nM Apt(ATP) + 100 mM NaCl solution and 5 mM MgCl_2_ at 25 °C

Apt(ATP)	Δ*G*° kcal/mol	Δ*H*° kcal/mol	Δ*S*° cal/(mol K)	*t*_m_ °C	*K*
Structure 1	− 1.7	− 22.30	− 69.09	49.6	17.7
Structure 2	− 1.31	− 25.70	− 81.80	41.0	9.14
Structure 3	− 1.06	− 26.90	− 86.67	37.2	5.99
Structure 4	− 0.99	− 23.30	− 74.83	38.2	5.32

Calculated using the UNAFold program

### Intracellular ATP determination via liposomal transfection of cancer cells with Apt(ATP)

**Fig. 3:**
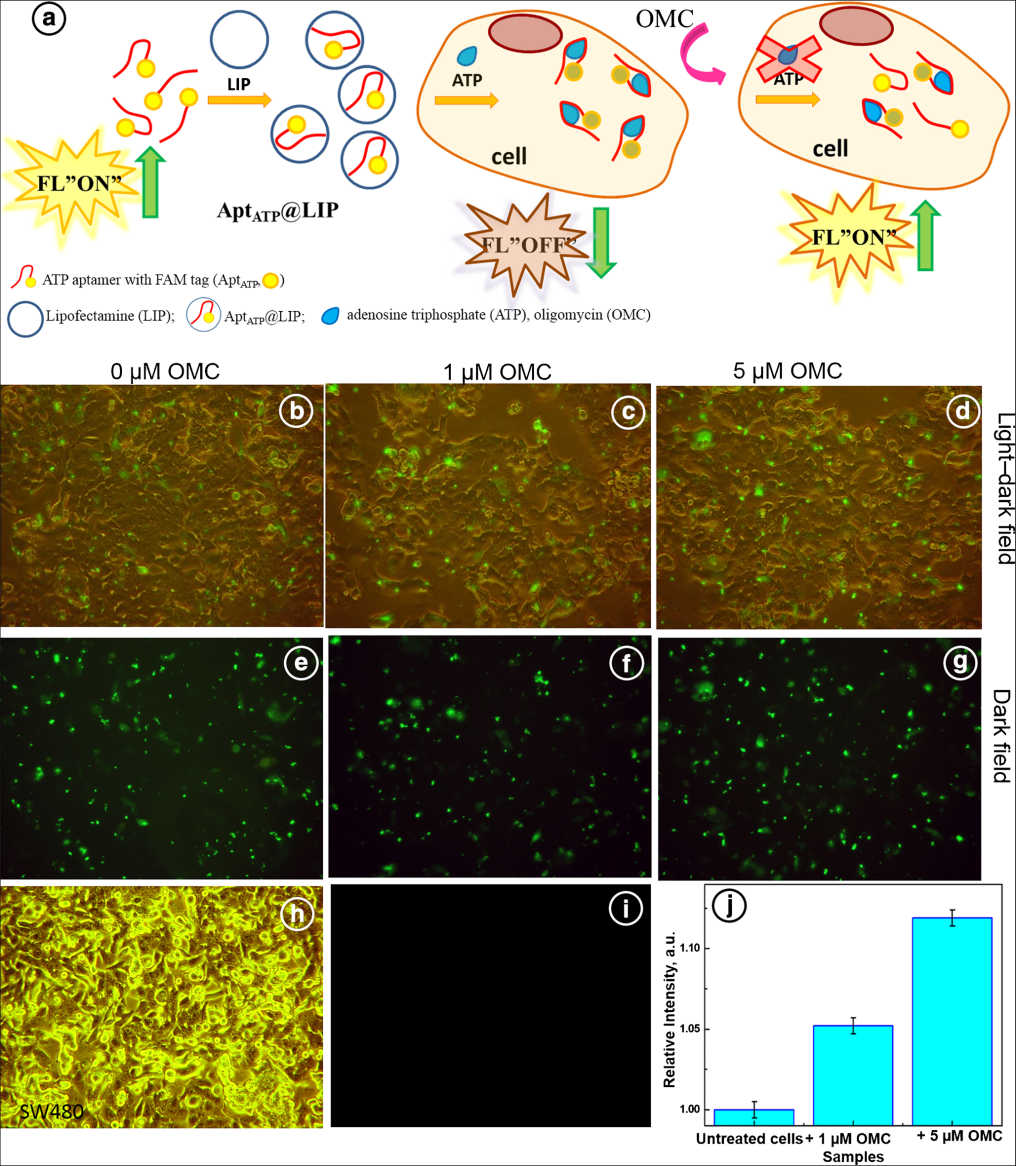
**a** Schematic view of the formation of Apt(ATP)@LIP for ATP recognition and ATP detection in cells. **b**–**g** Detection of ATP content in fixed SW480 cells untreated and treated with 1 and 5 μM concentration of oligomycin (OMC) for 20 h and transfected with Apt(ATP)@LIP for 135 min, **b**–**d** light-dark field, **e**–**g** dark field. **h** Morphology of the untreated SW480 cells. **i** Dark field fluorescence image of untreated SW480 cells (control). **j** Comparison of the relative intensity of fluorescence for SW480 cells transfected with Apt(ATP)@LIP after addition of OMC, *C*_OMC_ (μM) (1) 0, (2) 1.0, (3) 5.0, with incubation time *t*_inc_ = 20 h

Further investigations were focused on monitoring of the ATP synthase inhibition, modulated with oligomycin, in SW480 colorectal cancer cells, using ATP-sensing DNA aptamer. The decrease in ATP production due to the inhibition of ATP synthase can be readily discernible using the DNA aptamer. Simultaneous sensing and imaging of ATP in untreated and treated SW480 cells have been realized through inverted light microscope. A scheme of the cellular uptake of ATP aptamer with FAM fluorescence dye is presented in Fig. 3 a. In this scheme, a transfection of SW480 cells with Apt(ATP) using Lipofectamine carriers, Apt(ATP)@Lip, is shown. Thus, the ATP aptamer is delivered via a liposomal endocytosis to SW480 cells [60]. The Apt(ATP) with FAM fluorescence tag generates a strong green emission signal induced by the presence of intracellular ATP. The method applied was not targeting the organelles. Fluorescence probes for selective detection of ATP in mitochondria and lysosomes were investigated by Swamy et al. [51]. After entering the cell and interactions with ATP, inherently present in cytosol, a decrease in fluorescence signal of Apt(ATP) is observed due to the changes in conformation of Apt(ATP) and quenching of FAM dye (Fig. 3b–d). The interaction of cells with OMC causes a decrease of the production of ATP which is reflected in the observed increase of the aptamer fluorescence due to the weaker quenching at lower ATP concentration (Fig. 3f, g, j).

In Fig. 3, presented are cell images in the light-dark field (b–d) and in the dark field (e–g). The intensity of a green emission signal, related to the emission of FAM fluorophore, is correlated with Apt(ATP) introduced to cells after 135 min of incubation. It is seen that transfection with non-treated cells has shown the weakest green signal as compared with cells treated with oligomycin. The morphology of untreated SW480 cells is presented in Fig. 3 h and the dark field image of these untreated cells (control) is shown in Fig. 3 i, with no discernible fluorescence observed, as expected. After the interaction with OMC, the morphology of cells was not changed (data not shown). The data obtained in Fig. 3 b–g were confirmed by fluorescence measurements. The relative fluorescence signal of FAM of Apt(ATP) in transfected cells is presented in Fig. 3 j. The experiment with untreated cells (0 μM of OMC) was performed as a control. It is clearly seen that the addition of oligomycin at concentration 1 and 5 μM, respectively, per 20 h, is manifested by the increase of relative fluorescence signal about 5.2% and 11.9%, respectively. It indicates that the ATP production is diminished and therefore less binding between ATP and Apt(ATP) occurs and higher fluorescence signal of Apt(ATP) is observed. At the 0.05 level, these values are significantly different. Hao et al. [61] have shown that the treatment of H1299 cell line with oligomycin at concentrations of 100 and 1000 ng/mL results in a complete elimination of the respiration in 1 h. The authors have also shown that ATP levels were fully rebalanced within 4 h of the oligomycin treatment suggesting that the loss of oxidative phosphorylation (OXPHOS) of ATP was replaced with the increased glycolysis of ATP. Therefore, in our experiments, after 20 h of OMC treatment, the extra ATP production in SW480 cells by OXPHOS and glycolysis processes is likely to contribute to the non-linear dependence of Apt(ATP) signal on OMC concentration. The measured fluorescence signal of Apt(ATP) depends directly on ATP concentration.

### Modulation of oxygen consumption in cells with ATP synthase inhibitor oligomycin

**Fig. 4:**
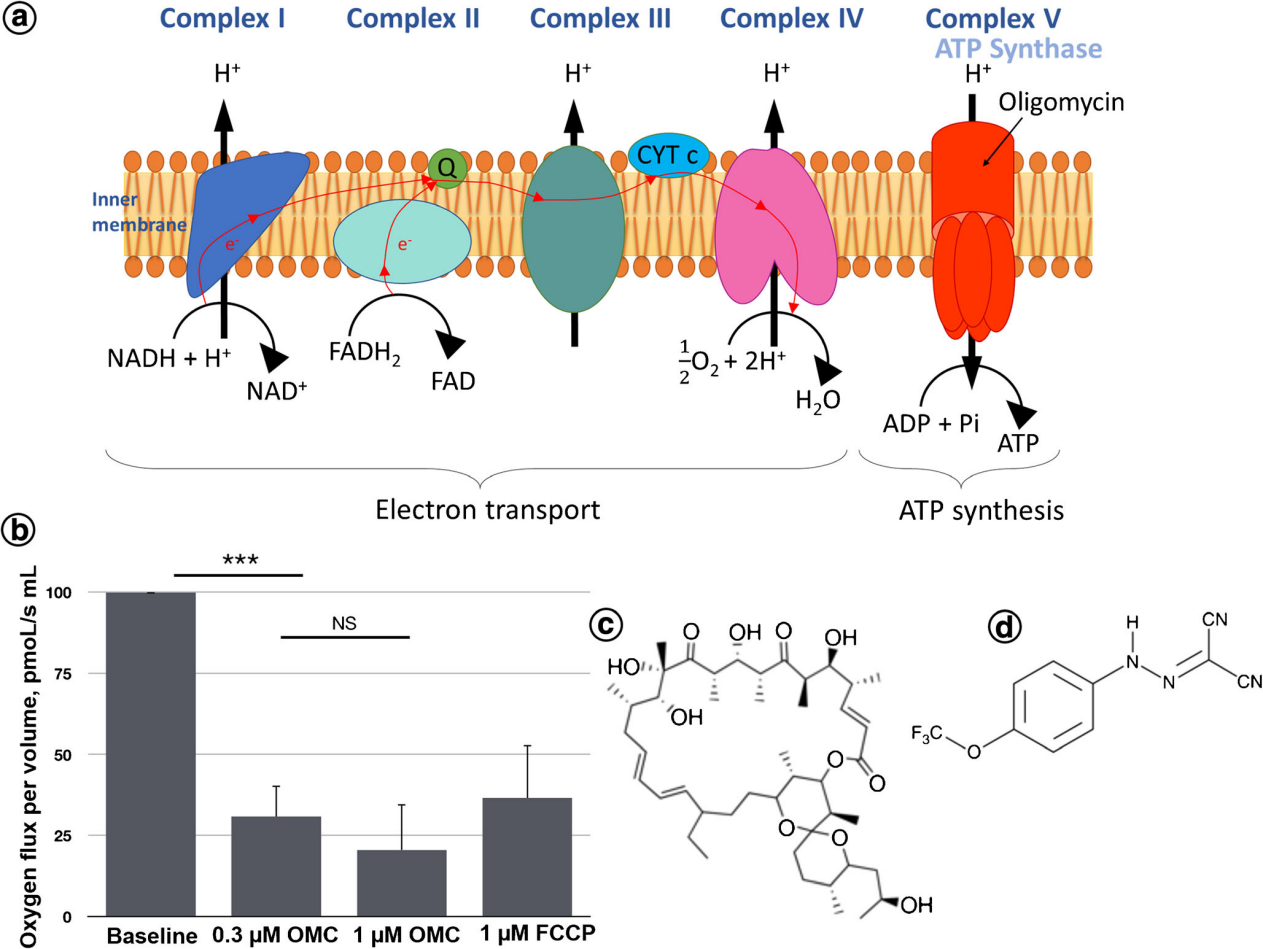
**a** Schematic illustration of mechanism of oligomycin (OMC) action in mitochondrial respiratory chain site. **b** Effect of OMC and FCCP on mitochondrial oxygen consumption reflecting ATP synthase activity in SW 480 cell line. **c** Structure of OMC. **d** Structure of FCCP. Conditions: *C*_OMC_ = 0.3 and 1 μM; *C*_FCCP_ = 1 μM, *n* = 3. ***Significantly different to baseline values with *p* < 0.01. NS—1 μM OMC values not significant to oligomycin 0.3 μM values

Data obtained by fluorescence measurements and microscopic images concerning the effect of oligomycin on mitochondrial ATP synthase and inhibition of ATP production were further confirmed by oxygen consumption in SW480 cells. In normal cells, ATP is mainly produced through oxidative phosphorylation (OXPHOS) in mitochondria and through glycolysis. However, in various cancer cells, the OXPHOS capacity is reduced and glycolysis is enhanced [62]. It is well known that oligomycin (OMC) is an inhibitor of mitochondrial F0 ATP synthase activity (Fig. 4a). OMC blocks the flow of protons. We have found that the application of 0.3 μM OMC to SW480 cells results in a threefold decrease in mitochondrial oxygen consumption rate, as calculated from the changes in oxygen concentration flux (Fig. 4b). For higher doses of oligomycin (1 μM), additional inhibition of respiration level was observed but value was not significant in comparison with oligomycin at 0.3 μM value, indicating that 0.3 μM OMC had the maximum effect. The mitochondrial oxygen consumption decreased from the control normalized baseline rate of 100 pmoL/(s mL) for untreated cells to 30.8 pmoL/(s mL) and 20.5 pmoL/(s mL) for 0.3 and 1 μM of oligomycin, respectively. The results were obtained immediately after OMC addition and indicate that the 20-h action of oligomycin on cells reported in previous experiments caused a total inhibition of ATP production. Interestingly, when a 1 μM mitochondrial oxidative phosphorylation uncoupler FCCP activator of proton conductance through the plasma membrane was applied, the activity of mitochondrial respiratory chain was partially restored and mitochondrial oxygen consumption increased to 36.6 pmoL/(s mL). Therefore, our experimental data confirm that oligomycin can be used to modulate the mitochondrial ATP synthase inhibition and ATP production. These data are supported by the Hao et al. studies performed on H1299 cancer cells [61]. Suppression of mitochondrial production of ATP due to OMC was also reported for SW620 cells by Lin et al. [63].

### Selectivity of the ATP aptamer-based fluorescence assay

**Fig. 5:**
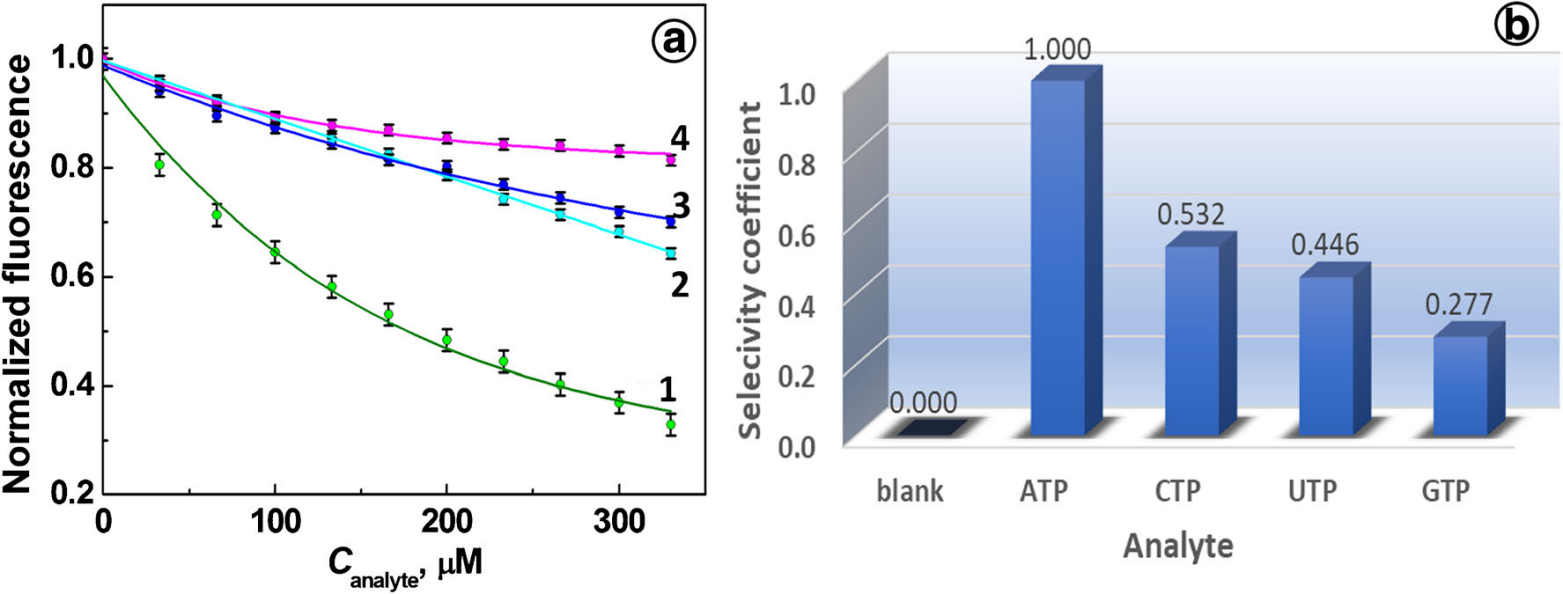
**a** Dependence of normalized fluorescence intensity of an ATP aptamer on concentration of nucleoside triphosphates: (1) ATP, (2) CTP, (3) UTP, (4) GTP. **b** Block diagram of selectivity coefficient for analytes *C*_Analytes_ = 330 μM. Conditions: *C*_Apt_ = 66.7 nM, 20 mM Tris-HCl buffer with 100 mM NaCl and 5 mM MgCl_2_, pH 7.4

The specificity of Apt(ATP) enables detection of ATP in complex biological samples. To evaluate the ATP aptamer selectivity against other nucleoside triphosphates such as GTP, CTP, and UTP, which are potential interferents, the selectivity tests were performed. The effect of the addition of nucleotides to a solution of Apt(ATP) is illustrated in Fig. 5 a. It is seen that addition of nucleoside triphosphates other than ATP with increasing concentrations results in weaker quenching of fluorescence emission of Apt(ATP) than that observed for ATP by iFRET. From the selected interferents, the weakest quenching was observed for GTP (18.6%) and strongest for CTP (35.7%), which compare favorably with that for ATP (67.1%).

The selectivity coefficients calculated for nucleoside triphosphates are shown in Fig. 5 b for 330 μM solutions of each triphosphate and 66.7 nM aptamer probe in 20 mM Tris-HCl saline buffer, pH 7.4. The values of selectivity coefficients, marked at each bar, range from 0.532 to 0.277.

### Cell viability decrease in OMC-treated cells

**Fig. 6:**
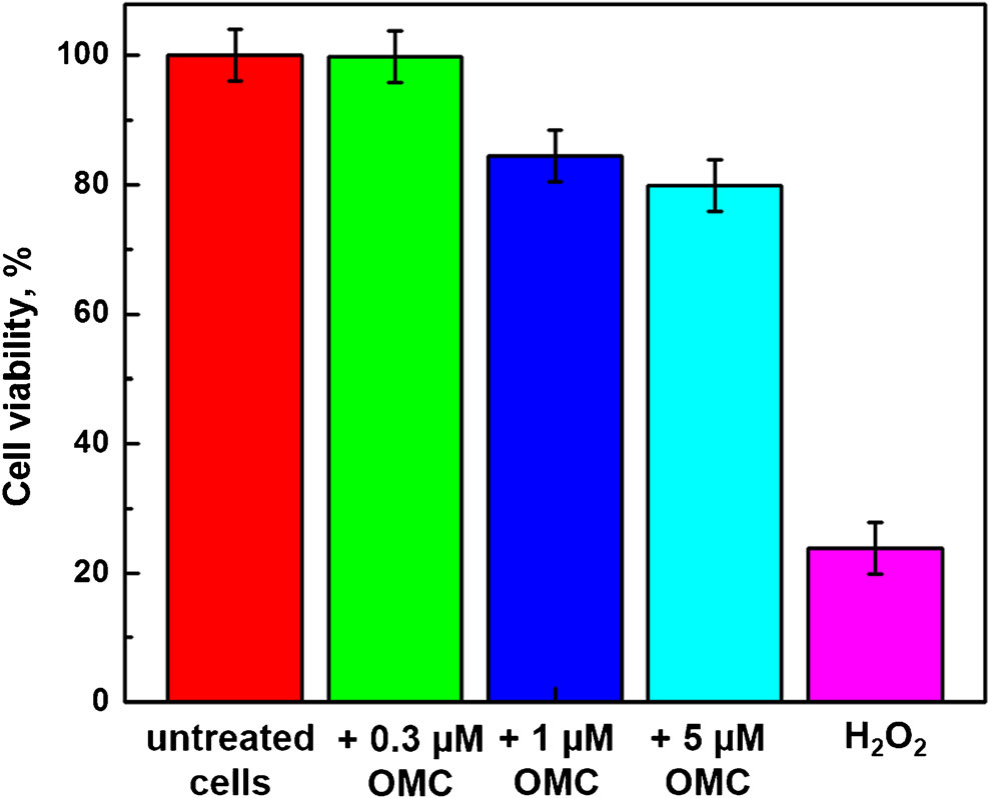
The results of MTT cell viability tests for cells treated with 0.3 μM, 1 μM, and 5 μM OMC; positive control: (OMC) = 0 μM; negative control: (H_2_O_2_) = 8 M

The effect of OMC treatment on cell viability was investigated using SW480 colon cancer cells. Since OMC inhibits ATP synthase, the cells treated with OMC are depraved of energy, and thus, their viability should be lower than that for untreated cells. The cell viability tests for untreated cells and those treated with OMC have been performed using a standard colorimetric MTT assay and the results are presented in Fig. 6. The test for untreated SW480 cells was used as the positive control and it shows 100% cell viability. It is seen that the addition of 0.3 μM of oligomycin for 20 h to SW480 cell line causes a negligible change in cell viability in comparison with control sample (untreated cells). The addition of oligomycin with 1 μM and 5 μM concentrations caused 15.5% and 20.1% decrease in cell viability, respectively. Lin and co-workers have shown that no obvious differences in cell viability between untreated SW620 cells and those treated with 12.6 and 25.2 μM OMC for 24 h were observed. They obtained 35.3% signal decrease with 25.2 μM OMC after a 48-h treatment [63]. In Fig. 6, we have also included a negative control with H_2_O_2_. A cell viability decrease of 76.1% was observed. These results are corroborated by recently published data. Tharakan’s group has shown that the blood-brain barrier endothelial cells treated with high concentration of H_2_O_2_ (> 10 mM) have shown a significant decrease in cell viability and induction of apoptosis [64]. Kaushik et al. have found that the reactive oxygen species (ROS) such as H_2_O_2_ generated by soft jet plasma and chemically induced ROS systems decreased the viability and intracellular ATP values of cancer T98G (glioblastoma), A549 (lung adenocarcinoma), normal HEK293 (embryonic kidney), and MRC5 (lung fibroblast) cells. Also, the ROS increased the apoptotic population [65].

### Sensing of ATP in cancer cell lysate

**Fig. 7:**
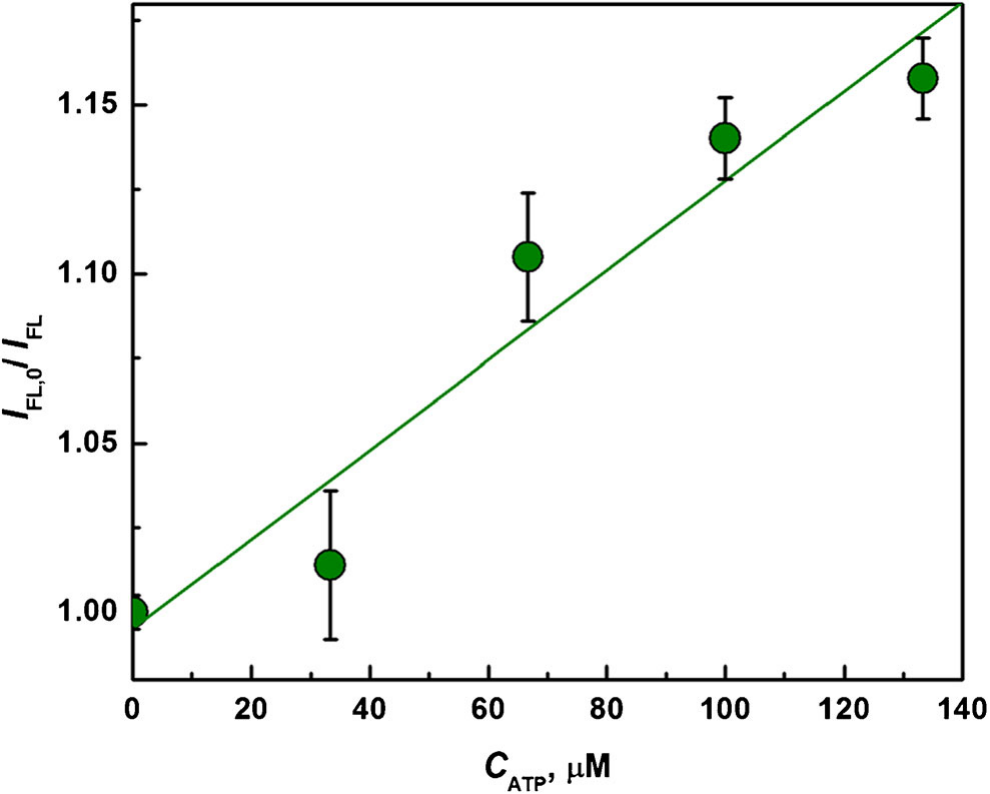
Test of Apt(ATP) sensitivity to the addition of ATP to a lysate solution of SW480 cells

The applicability of the proposed Apt(ATP) probe for the analysis of ATP in real samples was also tested using the cell lysate obtained by lysis of SW480 cancer cells. In Fig. 7, measurements of Apt(ATP) fluorescence upon the addition of ATP to the cell lysate diluted 1:300 in 20 mM Tris-HCl + 100 mM NaCl + 5 mM MgCl_2_ buffer are presented. The experiments were carried out using the diluted original lysate sample and samples spiked with 33, 66, 99, and 133 μM ATP (final added concentration). It is shown that the fluorescence signal of Apt(ATP) in lysate decreases by 16% for 133 μM ATP addition, confirming high sensitivity of the aptamer probe in 300-fold diluted cancer cell lysate. The high regression coefficient of the linear fitting to the experimental data, *R*^2^ = 0.964, indicates that the mechanism of intramolecular FRET remains operating in the lysate solution. The linear fitting equation is given by *I*_0_/*I* = 1 + 1.32 × 10^−3^*C*_ATP_ where *C*_ATP_ is given in (μM).

## Conclusions

The production of ATP is a key factor driving the life processes which strongly depend on energy supply. We have demonstrated that ATP production modulation by ATP synthase inhibitor, oligomycin, and the increased intracellular ATP level due to carcinogenesis can conveniently be monitored with a robust fluorescence “On-Off” switching aptamer probe, without any other added reagents or analyte labelling. We have successfully transfected living SW480 colon cancer cells with Apt(ATP), enabling fluorescence monitoring of intracellular ATP. The modulation of ATP production was correlated with oxygen consumption measurements in living cells and changes in cell viability tested using MTT method. Our results indicate that the Apt(ATP) has high selectivity toward ATP and good discrimination against non-specific interactions with cytosol and lysate components. Hence, the developed methodology may be applied in pharmacological studies and cancer research for convenient monitoring of dynamic ATP level modulation by new drugs under development and in the assessment of cancer growth rate.

## Electronic supplementary material

ESM 1 (PDF 258 kb)
