# Supplementary material for: Monitoring of dynamic ATP level changes by oligomycin-modulated ATP synthase inhibition in SW480 cancer cells using fluorescent “On-Off” switching DNA aptamer
Source: Anal Bioanal Chem. 2019 Aug 12;411(26):6899–911. doi: 10.1007/s00216-019-02061-0 (PMC6834760; doi:10.1007/s00216-019-02061-0)
Supplement: Supplementary file 1 — (PDF 258 kb) [file 216_2019_2061_MOESM1_ESM.pdf]

Analytical and Bioanalytical Chemistry

Electronic Supplementary Material

Monitoring of dynamic ATP level changes by oligomycin-modulated ATP synthase inhibition in SW480 cancer cells using fluorescent “On-Off” switching DNA aptamer

Katarzyna Ratajczak, Agnieszka Lukasiak, Hubert Grel, Beata Dworakowska,
Slawomir Jakiela, Magdalena Stobiecka

__Detection of ATP using fluorescent ATP aptamer (Apt(ATP))__

A response of an Apt(ATP) probe to the higher concentrations of ATP, in the mM range, is presented in Figure S1. The measurements under these conditions are characterized with a lower probe sensitivity, necessary to cover the larger dynamic range, including the elevated ATP levels in some cells under extreme conditions.

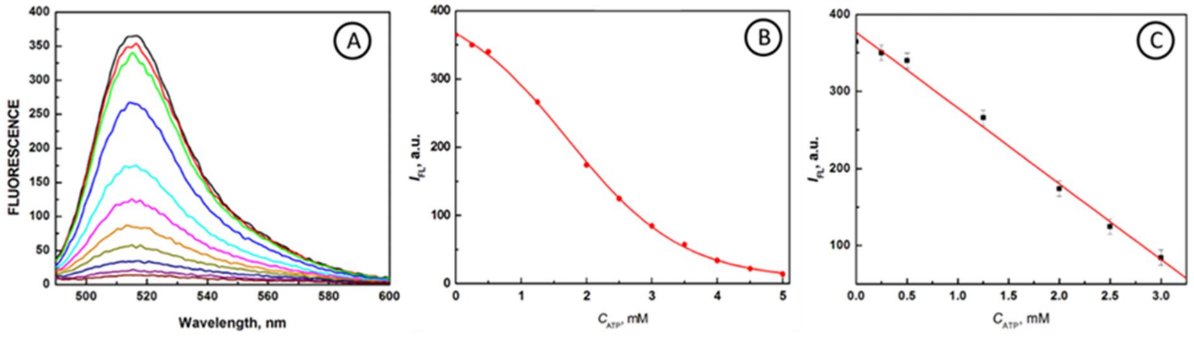

Fig. S1 (A) Fluorescence spectra for ATP aptamer after addition of ATP at different concentrations from 0 to 5 mM; (B) Dependence of I_{FL} vs. C_{ATP} in a wide range from 0 to 5 mM ATP; (C) Linear part of the dependence of I_{FL} vs. C_{ATP}
